# The effect of “typical case discussion and scenario simulation” on the critical thinking of midwifery students: Evidence from China

**DOI:** 10.1186/s12909-024-05127-5

**Published:** 2024-03-26

**Authors:** Yuji Wang, Yijuan Peng, Yan Huang

**Affiliations:** Department of Nursing, West China Second University Hospital, Sichuan University/West China School of Nursing, Sichuan University/Key Laboratory of Birth Defects and Related Diseases of Women and Children (Sichuan University), No. 20 Third Section, Renmin South Road, Chengdu, Sichuan Province 610041 China

**Keywords:** Medical education, Critical thinking, Nurse midwives

## Abstract

**Background:**

Assessment ability lies at the core of midwives’ capacity to judge and treat clinical problems effectively. Influenced by the traditional teaching method of “teacher-led and content-based”, that teachers involve imparting a large amount of knowledge to students and students lack active thinking and active practice, the clinical assessment ability of midwifery students in China is mostly at a medium or low level. Improving clinical assessment ability of midwifery students, especially critical thinking, is highly important in practical midwifery education. Therefore, we implemented a new teaching program, “typical case discussion and scenario simulation”, in the Midwifery Health Assessment course. Guided by typical cases, students were organized to actively participate in typical case discussions and to promote active thinking and were encouraged to practice actively through scenario simulation. In this study, we aimed to evaluate the effect of this strategy on the critical thinking ability of midwifery students.

**Method:**

A total of 104 midwifery students in grades 16–19 at the West China School of Nursing, Sichuan University, were included as participants through convenience sampling. All the students completed the Midwifery Health Assessment course in the third year of university. Students in grades 16 and 17 were assigned to the control group, which received routine teaching in the Midwifery Health Assessment, while students in grades 18 and 19 were assigned to the experimental group, for which the “typical case discussion and scenario simulation” teaching mode was employed. The Critical Thinking Disposition Inventory-Chinese Version (CTDI-CV) and Midwifery Health Assessment Course Satisfaction Questionnaire were administered after the intervention.

**Results:**

After the intervention, the critical thinking ability of the experimental group was greater than that of the control group (284.81 ± 27.98 and 300.94 ± 31.67, *p* = 0.008). Furthermore, the experimental group exhibited higher scores on the four dimensions of Open-Mindedness (40.56 ± 5.60 and 43.59 ± 4.90, *p* = 0.005), Analyticity (42.83 ± 5.17 and 45.42 ± 5.72, *p* = 0.020), Systematicity (38.79 ± 4.70 and 41.88 ± 6.11, *p* = 0.006), and Critical Thinking Self-Confidence (41.35 ± 5.92 and 43.83 ± 5.89, *p* = 0.039) than did the control group. The course satisfaction exhibited by the experimental group was greater than that exhibited by the control group (84.81 ± 8.49 and 90.19 ± 8.41, *p* = 0.002).

**Conclusion:**

The “typical case discussion and scenario simulation” class mode can improve the critical thinking ability of midwifery students and enhance their curriculum satisfaction. This approach carries a certain degree of promotional significance in medical education.

## Key points

- Typical case discussion and scenario simulation can improve midwifery students’ critical thinking ability.
- Typical case discussion and scenario simulation can enhance students’ learning interest and guide students to learn independently.
- Midwifery students were satisfied with the new teaching mode.

## Background

Maternal and neonatal health are important indicators to measure of the level of development of a country’s economy, culture and health care. The positive impact of quality midwifery education on maternal and newborn health is acknowledged in the publication framework for action strengthening quality midwifery education issued by the World Health Organization (WHO) [1]. Extensive evidence has shown that skilled midwifery care is crucial for reducing preventable maternal and neonatal mortality [2–4]. Clinical practice features high requirements for the clinical thinking ability of midwives, which refers to the process by which medical personnel analyze and integrate data with professional medical knowledge in the context of diagnosis and treatment as well as discover and solve problems through logical reasoning [5]. Critical thinking is a thoughtful process that is purposeful, disciplined, and self-directed and that aims to improve decisions and subsequent actions [6]. In 1986, the American Association of Colleges of Nursing formulated the “Higher Education Standards for Nursing Specialty”, which emphasize the fact that critical thinking is the primary core competence that nursing graduates should possess [7]. Many studies have shown that critical thinking can help nurses detect, analyze and solve problems creatively in clinical work and is a key factor in their ability to make correct clinical decisions [8–10].

However, the traditional teaching method used for midwifery students in China is “teacher-led and content-based”, and it involves efficiently and conveniently imparting a large amount of knowledge to students over a short period. Students have long failed to engage in active thinking and active practice, and the cultivation of critical thinking has long been ignored [5]. As a result, the critical thinking ability of midwifery students in China is mostly at a medium or low level [5]. Therefore, it is necessary to develop a new teaching mode to improve the critical thinking ability of midwifery students.

In 2014, Professor Xuexin Zhang of Fudan University, Shanghai, China, proposed a novel teaching method: the divided class mode. The basic idea of this approach is to divide the class time into two parts. The teachers explain the theoretical knowledge in the first lesson, and the students discuss that knowledge in the second lesson. This approach emphasizes the guiding role of teachers and encourages and empowers students to take responsibility for their studies [11]. Research has shown that the divided class mode can improve students’ enthusiasm and initiative as well as teaching effectiveness [12].

The problem-originated clinical medical curriculum mode of teaching was first established at McMaster University in Canada in 1965. This model is based on typical clinical cases and a problem-oriented heuristic teaching model [13]. The process of teaching used in this approach is guided by typical cases with the goal of helping students combine theoretical knowledge and practical skills. This approach can enhance the enthusiasm and initiative of students by establishing an active learning atmosphere. Students are encouraged to discuss and analyze typical cases to promote their ability to digest and absorb theoretical knowledge. Research has shown that the problem-originated clinical medical curriculum teaching mode can enhance students’ confidence and improve their autonomous learning and exploration ability. Scenario simulation teaching can provide students with real scenarios, allowing them to practice and apply their knowledge in a safe environment [14], which can effectively improve their knowledge and clinical skills and enhance their self-confidence [15, 16].

Based on the teaching concept of divided classes, our research team established a new teaching model of “typical case discussion and scenario simulation”. Half of the class time is allocated for students to discuss typical cases and carry out scenario simulations to promote their active thinking and active practice. The Midwifery Health Assessment is the final professional core course that midwifery students must take in our school before clinical practice. All students must complete the course in Grade 3. Teaching this course is important for cultivating the critical thinking and clinical assessment ability of midwifery students. Therefore, our team adopted the new teaching mode of "typical case discussion and scenario simulation" in the teaching of this course. This study explored the teaching mode’s ability to improve the critical thinking ability of midwifery students.

## Methods

### Study design

The study employed a semiexperimental design.

### Participants

A convenience sample of 104 third-year midwifery students who were enrolled in the Midwifery Health Assessment course volunteered to participate in this research at a large public university in Sichuan Province from February 2019 to June 2022 (grades 16 to 19). All the students completed the course in the third year of university. Students in grades 16 and 17 were assigned to the control group, which received the traditional teaching mode. Students in grades 18 and 19 were assigned to the experimental group, in which context the “typical case discussion and scenario simulation” class mode was used. The exclusion criteria for midwifery students were as follows: (1) dropped out of school during the study, (2) took continuous leave from school for more than two weeks, or (3) were unable to complete the questionnaire. The elimination criterion for midwifery students was that all the items were answered in the same way. No significant differences in students’ scores in their previous professional courses (Midwifery) were observed between the two groups. Textbooks, teachers, and teaching hours were the same for both groups.

### Development of the “typical case discussion and scenario simulation” class mode

This study is based on the implementation of the new century higher education teaching reform project at Sichuan University. With the support of Sichuan University, we first established a “typical case discussion and scenario simulation” class mode team. The author of this paper was the head of the teaching reform project and served as a consultant, and the first author is responsible for supervising the implementation of the project. Second, the teaching team discussed and developed a standard process for the “typical case discussion and scenario simulation” class mode. Third, the entire team received intensive training in the standard process for the “typical case discussion and scenario simulation” class mode.

### Implementation of the “typical case discussion and scenario simulation” class mode

#### Phase I (before class)

Before class, in accordance with the requirements for evaluating different periods of pregnancy, the teacher conceptualized typical cases and then discussed those cases with the teaching team and made any necessary modifications. After the completion of the discussion, the modified cases were released to the students through the class group. To ensure students’ interest, they were guided through the task of discovering and solving relevant problems using an autonomous learning approach.

#### Phase II (the first week)

Typical case discussion period. The Midwifery Health Assessment course was taught by 5 teachers and covered 5 health assessment periods, namely, the pregnancy preparation, pregnancy, delivery, puerperium and neonatal periods. The health assessment course focused on each period over 2 consecutive teaching weeks, and 2 lessons were taught per week. The first week focused on the discussion of typical cases. In the first lesson, teachers introduced typical cases, taught key knowledge or difficult evaluation content pertaining to the different periods, and explored the relevant knowledge framework. In the second lesson, teachers organized group discussions, case analyses and intergroup communications for the typical cases. They were also responsible for coordinating and encouraging students to participate actively in the discussion. After the discussion, teachers and students reviewed the definitions, treatments and evaluation points associated with the typical cases. The teachers also encouraged students to internalize knowledge by engaging in a process of summary and reflection to achieve the purpose of combining theory with practice.

#### Phase III (the second week)

Scenario simulation practice period. The second week focused on the scenario simulation practice period. In the first lesson, teachers reviewed the focus of assessment during the different periods and answered students’ questions. In the second lesson, students performed typical case assessment simulations in subgroups. After the simulation, the teachers commented on and summarized the students’ simulation evaluation and reviewed the evaluation points of typical cases to improve the students’ evaluation ability.

**Fig. 1:**
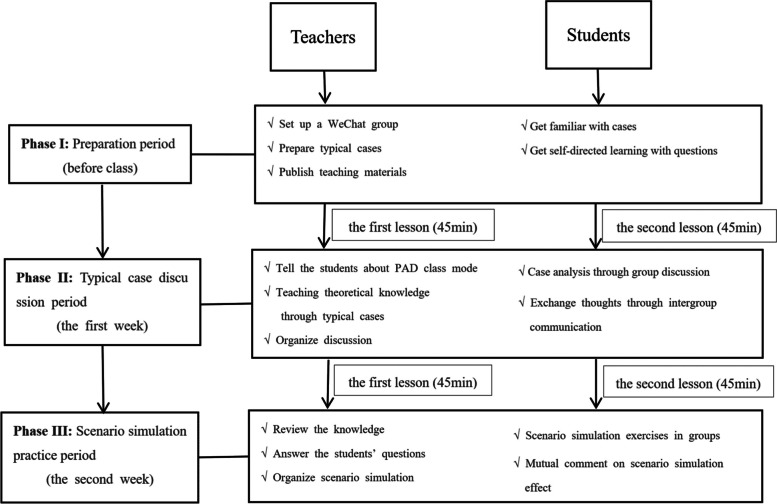
“Typical case discussion and scenario simulation” teaching mode diagram

The organizational structure and implementation of the “typical case discussion and scenario simulation” class mode showed in Fig. 1.

### Evolution

A demographic questionnaire designed for this purpose was used to collect relevant information from participants, including age, gender, single-child status, family location, experience with typical case discussion or scenario simulation and scores in previous professional courses (Midwifery).

The Critical Thinking Disposition Inventory-Chinese Version (CTDI-CV) was developed by Peng et al. to evaluate the critical thinking ability of midwifery students [17]. The scale contains 70 items across a total of seven dimensions, namely, open-mindedness, truth-seeking, analytical ability, systematic ability, self-confidence in critical thinking, thirst for knowledge, and cognitive maturity. Each dimension is associated with 10 items, and each item is scored on a 6-point Likert scale, with 1 indicating “extremely agree” and 6 representing “extremely disagree”. The scale includes 30 positive items, which receive scores ranging from “extremely agree” to “extremely disagree” on a scale of 6 to 1, and 40 negative items, which receive scores ranging from “extremely agree” to “extremely disagree” on a scale of 1 to 6. A total score less than 210 indicates negative critical thinking ability, scores between 211 and 279 indicate an unclear meaning, scores of 280 or higher indicate positive critical thinking ability, and scores of 350 or higher indicate strong performance. The score range of each trait is 10–60 points; a score of 30 points or fewer indicates negative trait performance, scores between 31 and 39 points indicate that the trait meaning is incorrect, scores of 40 points or higher indicate positive trait performance, and scores of 50 points or higher indicate extremely positive trait performance. The Cronbach’s α coefficient of the scale was 0.90, thus indicating good content validity and structure. The higher an individual’s score on this measure is, the better that individual’s critical thinking ability.

The evaluation of teaching results was based on a questionnaire used to assess undergraduate course satisfaction, and the researchers deleted and modified items in the questionnaire to suit the context of the “typical case discussion and scenario simulation” teaching mode. Two rounds of discussion were held within the study group to form the final version of the Midwifery Health Assessment satisfaction questionnaire. The questionnaire evaluates the effect of teaching in terms of three dimensions, namely, curriculum content, curriculum teaching and curriculum evaluation. The questionnaire contains 21 items, each of which is scored on a 5-point Likert scale, with 1 indicating “extremely disagree” and 5 representing “extremely agree”. The higher the score is, the better the teaching effect.

### Data collection and statistical analysis

We input the survey data into the “Wenjuanxing” platform (https://www.wjx.cn/), which specializes in questionnaire services. At the beginning of the study, an electronic questionnaire was distributed to the students in the control group via student WeChat and QQ groups for data collection. After the intervention, an electronic questionnaire was distributed to the students in the experimental group for data collection in the final class of the Midwifery Health Assessment course. All the data were collected by the first author (Yuji Wang). When students had questions about the survey items, the first author (Yuji Wang) immediately explained the items in detail. To ensure the integrity of the questionnaire, the platform required all the items to be answered before submission.

Statistical Package for Social Sciences Version 26.0 (SPSS 26.0) software was used for data analysis. The Shapiro‒Wilk test was used to test the normality of the data. The measurement data are expressed as the mean ± standard deviation (X ± S), and an independent sample t test was used for comparisons among groups with a normal distribution. The data presented as the number of cases (%), and the chi-square test was performed. A *P* value < 0.05 indicated that a difference was statistically significant.

### Ethical considerations

The study was funded by the New Century Teaching Reform Project of Sichuan University and passed the relevant ethical review. Oral informed consent was obtained from all individual participants in the study.

## Results

### Characteristics of the participants

A total of 104 third-year midwifery students were enrolled from February 2019 to June 2022, and 98.1% (102/144) of these students completed the survey. Two invalid questionnaires that featured the same answers for each item were eliminated. A total of 100 participants were ultimately included in the analysis. Among the participants, 48 students were assigned to the control group, and 52 students were assigned to the experimental group. The age of the students ranged from 19 to 22 years, and the mean age of the control group was 20.50 years (SD = 0.61). The mean age of the experimental group was 20.63 years (SD = 0.65). Of the 100 students who participated in the study, the majority (96.0%) were women. No significant differences were observed between the intervention and control groups in terms of students’ demographic information (i.e., age, gender, status as an only child, or family location), experience with scenario simulation or typical case discussion and scores in previous Midwifery courses (Table 1).

**Table 1:** Sociodemographic characteristics of the participants (*N* = 100)

Items		Control group (*n* = 48)	Intervention group (*n* = 52)	t	P
		Mean ± SD	Mean ± SD		
Age		20.50 ± 0.61	20.63 ± 0.65	1.052	0.295
The Midwifery course score		82.04 ± 3.16	82.71 ± 4.09	0.911	0.365
		N (%)	N (%)	X^2^	P
Gender	Male	1 (2.0)	3 (5.7)	0.883	0.347
	female	47 (98.0)	49 (94.3)		
Single-child status	YES	25 (52.0)	29 (55.7)	0.137	0.712
	NO	23 (48.0)	23 (44.3)		
Location of the family	Urban	34 (70.8)	35 (67.3)	0.145	0.703
	Rural	14 (29.2)	17 (32.7)		
Whether experienced PCMC	YES	46 (95.8)	50 (96.1)	0.007	0.935
or scenario simulation	NO	2 (4.2)	2 (3.9)		
The Midwifery course score					

### Examining the differences in critical thinking ability between the two groups

The aim of this study was to evaluate the effect of the new teaching mode of “typical case discussion and scenario simulation” on improving the critical thinking ability of midwifery students. Independent sample t tests were used to examine the differences in critical thinking ability between the two groups (Table 2). The results showed that the total critical thinking scores obtained by the experimental group were greater than those obtained by the control group (284.81 ± 27.98 and 300.94 ± 31.67, *p* = 0.008). The differences in four dimensions (Open-Mindedness (40.56 ± 5.60 and 43.59 ± 4.90, *p* = 0.005), Analyticity (42.83 ± 5.17 and 45.42 ± 5.72, *p* = 0.020), Systematicity (38.79 ± 4.70 and 41.88 ± 6.11, *p* = 0.006), and Critical Thinking Self-Confidence (41.35 ± 5.92 and 43.83 ± 5.89, *p *= 0.039)) were statistically significant.

**Table 2:** Comparison of critical thinking ability of Intervention group and Control group (*N* = 100)

Items	Control group (*n* = 48)	Intervention group (*n* = 52)	T	p
Truth-seeking	36.47 ± 6.01	36.65 ± 5.51	0.152	0.880
Open-mindedness	40.56 ± 5.60	43.59 ± 4.90	2.886	0.005*
Analyticity	42.83 ± 5.17	45.42 ± 5.72	2.366	0.020*
Systematicity	38.79 ± 4.70	41.88 ± 6.11	1.302	0.006*
Critical thinking self-confidence	41.35 ± 5.92	43.83 ± 5.89	2.819	0.039*
Inquisitiveness	44.72 ± 6.16	46.69 ± 6.80	1.507	0.135
Cognitive maturity	40.06 ± 7.90	42.86 ± 6.39	1.956	0.053
total	284.81 ± 27.98	300.94 ± 31.67	2.690	0.008*

*Means that the difference between the two groups was statistically significant

### Examining the differences in curriculum satisfaction between the two groups

To evaluate the effect of the new teaching mode of “the typical case discussion and scenario simulation” on the course satisfaction of midwifery students. Independent sample t tests were used to examine the differences in course satisfaction between the two groups (Table 3). The results showed that the curriculum satisfaction of the experimental group was greater than that of the control group (84.81 ± 8.49 and 90.19 ± 8.41, *p* = 0.002). Independent sample t tests were used to examine the differences in the three dimensions of curriculum satisfaction between the two groups (Table 3). The results showed that the average scores of the intervention group on the three dimensions were significantly greater than those of the control group (curricular content: 20.83 ± 1.96 and 22.17 ± 2.23, *p* = 0.002; curriculum teaching: 34.16 ± 3.89 and 36.59 ± 3.66, *p* = 0.002; curriculum evaluation: 29.81 ± 3.27 and 31.42 ± 3.19, *p* = 0.015).

**Table 3:** Comparison of curriculum satisfaction of Intervention group and Control group (*N* = 100)

Items	Control group (*n* = 48)	Intervention group (*n* = 52)	t	p
curriculum content	20.83 ± 1.96	22.17 ± 2.23	3.173	0.002*
curriculum teaching	34.16 ± 3.89	36.59 ± 3.66	3.212	0.002*
curriculum evaluation	29.81 ± 3.27	31.42 ± 3.19	2.287	0.015*
total	84.81 ± 8.49	90.19 ± 8.41	3.179	0.002*

*Means that the difference between the two groups was statistically significant

## Discussion

Midwifery is practical and intensive work. To ensure maternal and child safety, midwives must make decisions and take action quickly. Therefore, midwives should have both critical thinking ability and clinical decision-making ability [18]. In addition, the Australian Nursing and Midwifery Accreditation Council (ANMAC) regulates the educational requirements for the programs required for registration as a midwife. According to these standards, education providers must incorporate learning activities into curricula to encourage the development and application of critical thinking and reflective practice [19]. Therefore, the challenge of cultivating the critical thinking ability of midwifery students is an urgent problem that must be solved. However, influenced by the traditional teaching method of “teacher-led and content-based”, the critical thinking ability of midwifery students in China is mostly at a medium or low level. In order to improve the critical thinking ability of midwifery students. Our research team has established a new teaching model, the “typical case discussion and scenario simulation” class model. And applied to the midwifery core curriculum Midwifery Health Assessment. This study aimed to investigate the implementation of a novel systematic and structured teaching model for midwifery students and to provide evidence regarding how to improve the critical thinking ability of midwives.

The results showed that the total CTDI-CV score obtained for the experimental group was greater than that obtained for the control group. These findings indicate that the “typical case discussion and scenario simulation” class mode had a positive effect on the cultivation of students’ critical thinking ability, a conclusion which is similar to the findings of Holdsworth et al. [20], Lapkin et al. [21] and Demirören M et al. [22]. We indicate the following reasons that may explain these results.The core aim of the typical case discussion teaching mode is to raise questions based on typical clinical cases and to provide heuristic teaching to students [23]. This approach emphasizes asking questions based on specific clinical cases, which enables students to engage in targeted learning. Moreover, scenario simulation allows students to attain certain inner experiences and emotions and actively participate in curriculum practice, which can enhance their ability to remember and understand knowledge [24]. Through the divided class mode, half of the class time was divided into the students. This method emphasizes the guiding role of teachers and encourages and empowers students to assume learning responsibilities. In addition, students can think, communicate and discuss actively [22, 23]. Furthermore, this approach created opportunities for students to analyze and consider problems independently and give students sufficient time to internalize and absorb knowledge and deepen their understanding of relevant knowledge, which can increase their confidence in their ability to address such problems and improve their critical thinking ability [12, 25, 26].

In addition, the results showed that except for Truth-Seeking and Systematicity, the other five dimensions were all positive. These findings are similar to the results reported by Atakro et al.. and Sun et al. [27, 28]. Through the intervention, the Systematicity scores became positive, suggesting that the new teaching mode can help students deal with problems in an organized and purposeful way. However, Truth-Seeking still did not become positive; this notion focuses on intellectual honesty, i.e., the disposition to be courageous when asking questions and to be honest and objective in the pursuit of knowledge even when the topics under investigation do not support one’s self-interest [29]. Studies have shown that this factor is related to the traditional teaching mode used [30]. The traditional teaching mode focuses on knowledge infusion, helps students remember the greatest possible amount of knowledge in a short time, and does not focus on guiding students to seek knowledge with sincerity and objectivity. Therefore, in future educational practice, we should focus on cultivating students’ ability to seek truth and engage in systematization.

Student evaluative feedback is an important way to test the effectiveness teaching mode. Therefore, understanding students’ evaluations of the effects of classroom teaching is key to promoting teaching reform and improving teaching quality. Therefore, we distributed a satisfaction questionnaire pertaining to the midwifery health assessment curriculum, which was based on the “typical case discussion and scenario simulation” class mode, with the goal of investigating curriculum satisfaction in terms of three dimensions (curriculum content, curriculum teaching and curriculum evaluation). The results showed that the satisfaction scores for each dimension increased significantly. This finding suggests that the new teaching method can enrich the teaching content, diversify the teaching mode and improve students’ curriculum evaluations.

In summary, the “typical case discussion and scenario simulation” class mode focuses on typical cases as its main content. Students’ understanding of this content is deepened through group discussion and scenario simulation. The subjectivity of students in curriculum learning should be accounted for. Students can be encouraged to detect, analyze and solve problems with the goal of improving their critical thinking ability. Moreover, this approach can also enhance curriculum satisfaction. It is recommended that these tools should be used continuously in future curriculum teaching.

This study has several limitations. First, the representativeness of the sample may be limited since the participants were recruited from specific universities in China. Second, we used historical controls, which are less effective than simultaneous controlled trials. Third, online self-report surveys are susceptible to response biases, although we included quality control measurements in the process of data collection. Fourth, we did not use the same critical thinking instrument, CTDI-CV, to investigate the critical thinking of the students in the experimental group or the control group before intervention but used professional course grades from the Midwifery for substitution comparison. This may not be a sufficient substitute. However, these comparisons could be helpful since those grades included some sort of evaluation of critical thinking. In light of these limitations, future multicenter simultaneous controlled studies should be conducted. Nonetheless, this study also has several strengths. First, no adjustment of teachers or change in learning materials occurred since the start of the midwifery health assessment, thus ensuring that the experimental and control groups featured the same teaching materials, teachers and teaching hours. In addition, to ensure the quality of the research, the first author of this paper participated in the entirety of the course teaching.

## Conclusion

The “typical case discussion and scenario simulation” class mode can improve the critical thinking of midwifery students, which is helpful for ensuring maternal and child safety. Students are highly satisfied with the new teaching mode, and this approach has a certain degree of promotional significance. However, this approach also entails higher requirements for both teachers and students.

## Data Availability

The datasets used and/or analyzed during the current study are available from the corresponding author on reasonable request.
